# Surgeon-Delivered Nerve Block for Reduction of Perioperative Pain and Opioid Use After Lumbosacral Spine Surgery

**DOI:** 10.1001/jamanetworkopen.2022.48439

**Published:** 2022-12-27

**Authors:** Evan F. Joiner, Justin A. Neira, Wisdom E. Yevudza, Mark A. Weller, Gebhard Wagener, Peter D. Angevine, Christopher E. Mandigo

**Affiliations:** 1 Department of Neurological Surgery, Columbia University-NewYork Presbyterian Hospital, New York; 2 Department of Anesthesiology, Hospital for Special Surgery, New York, New York; 3 Department of Anesthesiology, Columbia University-NewYork Presbyterian Hospital, New York

## Abstract

This cohort study compares postoperative pain scores, opioid use, and length of hospital stay among adults undergoing noninstrumented lumbosacral surgery who received x-ray–guided dorsal ramus block vs those who did not.

## Introduction

Over 900 000 US adults undergo spine surgery annually.^1^ Their postoperative pain is difficult to manage, often requiring high-dose opioids. There is a need for surgeons to collaborate to develop therapies that improve postoperative pain management and reduce perioperative opioid use. Although surgeon-delivered blocks have been successfully used in joint replacement surgery,^2^ limited data exist on surgeon-performed nerve blocks for spine surgery.^3^ This cohort study evaluated use of a novel surgeon-delivered x-ray–guided dorsal ramus block (XDRB) for noninstrumented lumbar surgery.

## Methods

We analyzed a retrospective cohort of all consecutive patients who underwent noninstrumented lumbosacral surgery with 2 neurosurgeons (C.E.M., P.D.A.) in a tertiary care center between February 2020 and March 2021 (eMethods in Supplement 1). Patients received total intravenous anesthesia with or without radiography-guided bupivacaine injections targeted to the dorsal rami of spinal nerves (XDRB) (Figure). Primary outcomes were first visual analog scale (VAS) pain score in the postanesthesia care unit (PACU), PACU opioid use, and length of stay. Means for each outcome were compared between treatment groups and assessed for differences using an unpaired 2-sample *t* test. A 2-sided *P* < .05 was considered statistically significant. Data analysis was performed using Stata 15.1 (StataCorp LLC). The Columbia University Institutional Review Board approved this study and waived the informed consent requirement because the study posed minimal risk to participants. We followed the STROBE reporting guideline.

**Figure.**
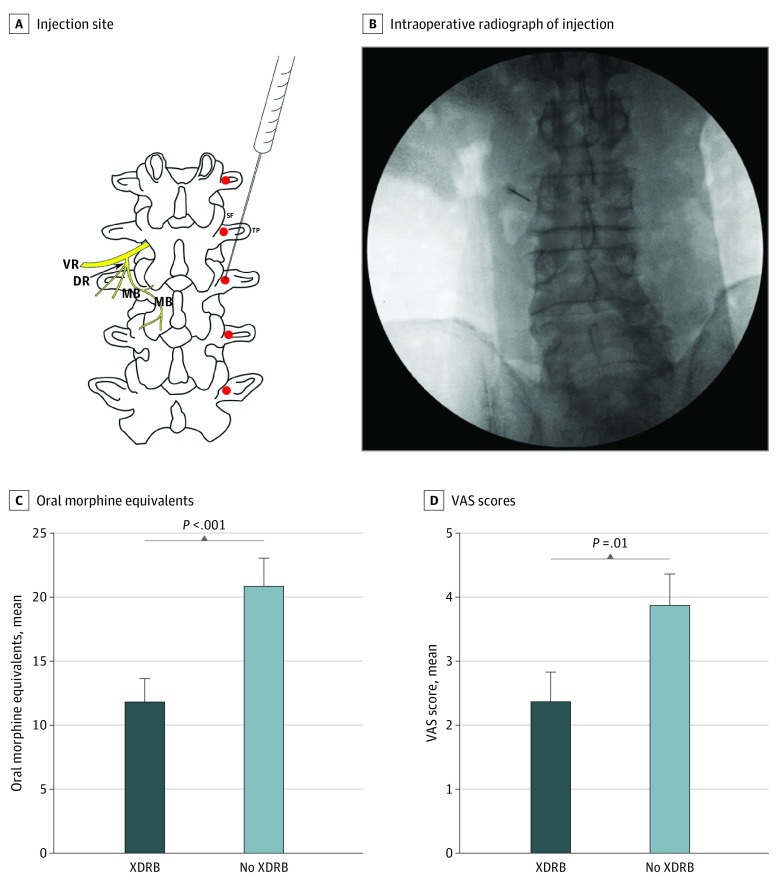
Injection Site and Comparison of Morphine Requirements and Pain Scores in Patients With and Without X-ray–Guided Dorsal Ramus Block A, X-ray–guided dorsal ramus block (XDRB) injection at junction of L3 superior facet and transverse process. Lumbar nerve root is shown with ventral ramus (VR) and dorsal ramus (DR) and medial branch (MB) of DR. B, Intraoperative XDRB being placed at junction of left superior facet and transverse process at L3. Volume of injection at each level (2.5-5.0 cc), strength of formulation of bupivacaine (0.25%-0.5%), number of blocked levels, and laterality of block were determined at the discretion of the performing surgeon. C and D, Comparison of opioid requirements and visual analog scale (VAS) pain scores between XDRB (n = 58) and non-XDRB (n = 49) groups. Error bars represent the SE of the mean.

## Results

We included 107 patients, of whom 58 underwent XDRB before surgery (33 males [56.9%], 25 females [43.1%]; mean [SD] age 58.8 [15.6] years) and 49 received no XDRB (31 males [63.3%], 18 females [36.7%]; mean [SD] age 61.7 [15.7] years) (Table). These treatment groups were similar.

**Table.** Baseline Characteristics of XDRB and Non-XDRB Groups

Characteristic	No./total No. (%)	
	XDRB group (n = 58)	Non-XDRB group (n = 49)
Age, mean (SD), y	58.8 (15.6)	61.7 (15.7)
Sex		
Male	33/58 (56.9)	31/49 (63.3)
Female	25/58 (43.1)	18/49 (36.7)
Extent of surgery, mean (SD), spinal levels	1.32 (0.60)	1.35 (0.75)
Type of surgery		
Decompression and/or discectomy	54/58 (93.1)	42/49 (85.7)
Intradural tumor resection or tethered cord release	3/58 (5.2)	1/49 (2.0)
Removal of hardware	1/58 (1.7)	1/49 (2.0)
Wound washout	0/58	5/49 (10.2)
Incisional infiltration with local anesthetic		
Yes	58 (100.0)	39/49 (79.6)
No	0/58	10/49 (20.4)
Intraoperative long-acting opioid medications (fentanyl, sufentanil, dilaudid), mean (SD), OME	50.6 (3.8)	56.8 (4.5)

Abbreviations: OME, oral morphine equivalents; XDRB, x-ray–guided dorsal ramus block.

Patients in the XDRB group had mean first PACU VAS scores that were 1.5 (of 10) points lower than those in the non-XDRB group (2.4 vs 3.9; 39% reduction; 95% CI, −0.3 to −2.8; *P* = .02) and required, on average, 9.0 fewer oral morphine equivalents (OMEs) in PACU (12.0 vs 21.0; 43% reduction; 95% CI, −3.8 to −14.2; *P* < .001) (Figure). All patients in the XDRB group received incisional infiltration with local anesthetic compared with 79.6% of patients in the non-XDRB group. After excluding patients who did not receive infiltration, patients in the XDRB group still had mean VAS scores that were 1.7 points lower than those in the non-XDRB group (2.4 vs 4.1; 95% CI, −0.4 to −3.1; *P* = .01) and required, on average, 9.6 fewer OMEs (12.0 vs 21.6; 95% CI, −4.0 to −15.2; *P* < .001). Despite moderately skewed data for both variables, sample size was sufficient by the central limit theorem to apply the *t* test.

There was no difference in mean length of stay between the XDRB and non-XDRB groups (1.1 vs 1.5 days; 95% CI, −0.9 to 1.7 days; *P* = .56). Mean (SD) time required to perform XDRB was 5.9 (2.6) minutes. No adverse events occurred in either group.

## Discussion

Novel surgeon-delivered XDRB was associated with lower postoperative pain scores and decreased opioid use. The mean difference in VAS score between groups was greater than the minimal clinically important difference for acute postoperative VAS score (0.99 of 10).^4^

Acute postoperative opioid exposure represents an important risk factor for opioid abuse.^5,6^ Local anesthetic nerve blocks represent a key strategy to reduce perioperative pain and opioid use. While progress has been made in developing ultrasonography-guided nerve blocks delivered by anesthesiologists, including erector spinae plane and thoracolumbar interfascial plane blocks, XDRB shifts the local anesthetic block from a distinct preoperative procedure performed by an anesthesiologist to an intraoperative surgeon-delivered entity.

The study was limited by its small sample size and retrospective, nonrandomized design. Randomized clinical trials are warranted to evaluate XDRB efficacy. Optimization of pain control through use of surgeon-delivered local anesthetic blocks may represent an opportunity, in various surgical fields, to improve patient recovery, reduce postoperative opioid use, and decrease opioid abuse.

## Notes

Supplement 1.
eMethods.

Supplement 2.
Data Sharing Statement
