# Supplementary material for: Surgeon-Delivered Nerve Block for Reduction of Perioperative Pain and Opioid Use After Lumbosacral Spine Surgery
Source: JAMA Netw Open. 2022 Dec 27;5(12):e2248439. doi: 10.1001/jamanetworkopen.2022.48439 (PMC9857272; doi:10.1001/jamanetworkopen.2022.48439)
Supplement: Supplement 1. — eMethods. [file jamanetwopen-e2248439-s001.pdf]

Supplementary Online Content

Joiner EF, Neira JA, Yevudza WE Jr, et al. Surgeon-delivered nerve block for reduction of perioperative pain and opioid use after lumbosacral spine surgery. *JAMA Netw Open*. 2022;5(12):e2248439. doi:10.1001/jamanetworkopen.2022.48439

eMethods.

This supplementary material has been provided by the authors to give readers additional information about their work.

© 2022 Joiner EF et al. *JAMA Network Open.*

eMethods.

This retrospective cohort study was performed in keeping with STROBE reporting guidelines for cohort studies, and a STROBE checklist was submitted to the editorial board for review. Patient characteristics including age, sex, extent of surgery, type of surgery, use of incisional infiltration with local anesthetic, and use of intraoperative long-acting opioid medications were compared between both groups. Of note, total amount of long-acting opioid medications (specifically fentanyl, sufentanil, and dilaudid) dosed within the operating room was calculated and converted to oral morphine equivalents. Dosing of fast-acting opioid medication (remifentanil) was not included in this total under the assumption that remifentanil is cleared quickly enough that all analgesic effects of the drug have abated by the time a patient leaves the operating room. If the groups differed substantially on one of the above characteristics, then stratified analysis was performed in order to evaluate for potential confounders. If full stratified analysis was not possible due to limited number of patients with a given characteristic, then sensitivity analysis was performed based on exclusion of the relevant patients.

© 2022 Joiner EF et al. *JAMA Network Open.*
