# Supplementary material for: Surgeon-Delivered Nerve Block for Reduction of Perioperative Pain and Opioid Use After Lumbosacral Spine Surgery
Source: JAMA Netw Open. 2022 Dec 27;5(12):e2248439. doi: 10.1001/jamanetworkopen.2022.48439 (PMC9857272; doi:10.1001/jamanetworkopen.2022.48439)
Supplement: Supplement 2. — Data Sharing Statement [file jamanetwopen-e2248439-s002.pdf]

Data Sharing Statement

Joiner. Surgeon-Delivered Nerve Block for Reduction of Perioperative Pain and Opioid Use After Lumbosacral Spine Surgery. *JAMA Netw Open*. Published December 27, 2022. doi:10.1001/jamanetworkopen.2022.48439

Data
Data available: Yes
Data types: Deidentified participant data, Data (not involving human participants), Data dictionary
How to access data: Data available upon request by contacting Dr. Evan F. Joiner, MD at efj2107@cumc.columbia.edu.
When available: With publication

Supporting Documents
Document types: Statistical/analytic code
How to access documents: Supporting documents available upon request by contacting Dr. Evan F. Joiner, MD at efj2107@cumc.columbia.edu.
When available: With publication

Additional Information
Who can access the data: Anyone requesting the data.
Types of analyses: For any purpose.
Mechanisms of data availability: Data will be made available after approval of a proposal.
